# Supplementary material for: Data-driven reduced modeling of neural dynamics
Source: Nat Commun. 2026 Jul 28;17:9123. doi: 10.1038/s41467-026-75924-7 (PMC13519027; doi:10.1038/s41467-026-75924-7)
Supplement: Supplementary file 2 [file 41467_2026_75924_MOESM2_ESM.pdf]

nature portfolio

Peer Review File

Data-Driven Reduced Modeling of Neural Dynamics

Corresponding Author: Ms Alice Marraffa

This file contains all reviewer reports in order by version, followed by all author rebuttals in order by version.

Version 0:

Reviewer comments:

Reviewer #1

(Remarks to the Author)
Referee Report for 'Data Driven Reduced Modeling ….'' By Marraffa et al.

This was an interesting paper to read, in that it brings together phenomenology (and terminology) from two "domain sciences" --neuroscience and dynamical systems—in ways that, I believe, would be beneficial to both sides. The meaningful interpretation of, for example, Ref. 18, and the clear presentation/discussion/interpretation of line and ring attractors is good reading (for me, and, I expect, for several people on the dynamical systems side). The SSM (spectral submanifold) "technology" developed and deployed by Prof. Haller is a meaningful approach to quantifying and interpreting slow reduced dynamics, and as such, it is useful conceptually (in the directions discussed) and practically (in the examples discussed, which I consider as well chosen). To the best of my ability to evaluate them, the results are correct and well presented.
In terms of recommending the paper for publication, I have two thoughts to offer.
The first considers the paper as a meaningful educational demonstration – providing nontrivial (the word nontrivial is important!) mathematical caricatures of types of neuronal activity (e.g. decision making, working memory). Instead of working with a "completely toy" model of two, three, five neurons that exhibit a mathematical analog to these behaviors – which should be possible, maybe somebody has done it—the paper goes beyond that to models of, say, O(100) neurons. There, the SSM reduction techniques developed by Prof. Haller become relevant: one can still usefully computationally approximate "slow" low-dimensional manifolds associated with the medium- and long term dynamics of the problem – observe them, analyze them and interpret them. In that sense, the paper is indeed of interest to several readers.
A quick "transitional" comment: I either disagree or misinterpret the statement on page 2 that "identifying such dynamical motifs requires accurate models of neural dynamics" – I am convinced that the phenomena are there for many, many different neuronal ODE models (I am sure we agree on that with the authors). The statement probably refers to the ability to quantitatively identify these behaviors.
This comment brings me to my second thought about the recommendation of the paper for publication. It is important to say, before I start, that what I am saying now is a personal opinion about the modeling of such physiological systems, that I can argue about, but since I am a "dynamical systems modeler with little experience in computational neuroscience" and not an actual neuroscientist, I can offer it only as a personal opinion.
The authors work with actual steady states / limit cycles, and slow submanifolds of them, for systems of say O(100) differential equations. The important thing here for me is NOT the number 100 (it could be 1000, or 10,000) BUT the assumption that we have an ACTUAL steady state of that system, so we can compute its linearization (and then go on to computations of slow manifolds starting from its neighborhood). If that is accurate physically (if in neuroscience we have ACTUAL steady states of the equations for the neurons) then everything about the paper is OK.
If, however, as I suspect, we do NOT have actual steady states for large ensembles of neurons, but we rather have some sort of "statistical steady state" or "coarse grained steady state" or "statistical slow state" of the behavior, then I see a problem – the dynamical equations whose steady states we should work with are not the exact steady states of the 100 neurons, but rather some low-dimensional effective, coarse grained equations for the ensemble – a "closed" set of effective equations for the behavior obtained somehow (theory-driven, data-driven) for the 100 or 1000 detailed dynamical neurons – which might never have an actual steady state at all, but rather, say, a local "ball" of small-scale messy/stochastic/chaotic behavior that can be thought of as a coarse-grained effective activity steady state.
IF this option is true, then the paper does not discuss obtaining the effective coarser equations on which the SSM technology should be applied – it only talks about exact, detailed steady states/ limit cycles of the 100 or 1000 detailed neuron dynamical equations.
If my view of the physiological situation is true, then a discussion of "how we get the equations on which to do SSM" is vital, and at the moment missing from the paper – and it should be meaningfully added.

If the physiological situation involves actual steady states of 100 or 1000 neuronal model ODEs, or if the paper is talking about finite RNN model themselves, and not so much about the physiological situation, then the paper could be published as a dynamical reduction case study; yet I think the authors are more ambitious than that in their presenting and discussing connection with physiological neuroscience.
SO: if there is a need for "effective" steady states and "effective" equations, this should be clarified and discussed. If I am wrong, and there is no need for "effective steady states" then the paper is OK as is.

(Remarks on code availability)

Reviewer #2

(Remarks to the Author)
The manuscript is an ambitious and substantial work aimed at bolstering the authors' spectral submanifold (SSM) theory by demonstrating reductions of dynamical recurrent neural networks proposed in recent publications on learning. The theory is mathematically somewhat mature, with several publications giving rigorous proofs of existence and uniqueness of spectral manifolds emanating from fixed points that generalize the well-known stable and unstable manifolds and separatrices emanating from hyperbolic fixed points. Furthermore, in previous publications, the authors have developed a data-driven approach for calculating these SSMs, enabling their approximation for systems whose governing equations are not known.

The content and presentation style are somewhat unusual for a broad-audience journal. Broadly, I felt that the methods and supplementary information together were basically a long-format technical article, and the main text was an effort to compress that information into the journal's more constrictive article format. Consequently, the main text may be hard to follow for an average reader who lacks a strong dynamical systems background.

Another small complaint that I have is that the class of "recurrent neural networks" encapsulated by the coupled ODEs in Eq. (14) is rather specific to the dynamical systems framework, which might be considered unconventional. Many artificial neural networks do not really have a temporal variable at all. The models and slow point views described in the introduction on p3 are more narrowly focused on a specific class of biological systems and neuronal dynamics, so much so that I felt that a more descriptive title for the manuscript would be "Data-Driven Reduced Modeling of Recurrent Neuronal Dynamics." An overly cynical reader may conclude that the manuscript is the work of mathematicians who cherry-picked applied research to find evidence of impactful work that would support their preferred theory.

As for a recommendation of whether the manuscript is suitable for the journal, I think I disappointingly have to say that the decision is above my pay grade. I enjoyed reading the manuscript and found it educational and interesting. I was left a little skeptical about how impactful the results would be to a broader audience, including the few among the developers of the models studied. Nevertheless, it stirred some inspirational thoughts in my mind. For example, in the study partial differential equations, symmetries in the jet space approach [Olver, Applications of Lie groups to differential equations. Springer, 1993] give rise to self-similar solutions that generalize fixed points in an appropriate coordinate system of invariants (see, e.g. the rather obscure and inconsequential [Nicolaou, Symmetry and variational analyses of fluid interface equations in the thin film limit, 2017]). Could the SSM approach say anything broadly about the development of turbulence, or could the data-driven approximations give rise to new approaches?

Other more minor notes I recorded while reading:
1. What exactly is the goal of producing a reduced-order model for an RNN? To replace the RNN with a less expensive surrogate? Or to make some more interpretable model to show how learning works?
2. Figure captions in the main text are huge, and panels aren't referenced individually and in order that they appear in the main text, making them a little impenetrable. Similarly, I'd prefer that the sections of the supplement and the methods were referenced in the main text where first relevant, so that it was possible to read just the main text alone to understand the most important message and reference methods and the supplement only if/when desired.
3. For readers hoping to use the SSMLearn package without strong dynamical systems expertise, more practical guidance would be helpful for a broad-audience publication. How does one select the dimension d of the manifold and the order of the Taylor series approximation? Can you provide a score for the fit on a test dataset and a Pareto-front or AIC criterion for the selection?
4. Fig. 5: are there typos in the caption? The sine wave is on the right and the context choice is left?
5. The future tense at the end of the methods sections, e.g. pg 24 "We will treat this discontinuous…" is odd. If the supplement is truly supplementary, the reader should be able to read the main text without worrying about what will be done in the supplement. It would be better to note that "We treat this discontinuous…" as a part of the discussion in the main text.
6. Creating a recurrent neural network to generate a sine wave is certainly an odd goal without some context. It only takes two real variables to describe a simple harmonic oscillator—why introduce a bulky neural network?
7. Supplement, p3: "inverient" is a typo. Also, the paragraph before S1 is pretty repetitive given the main-text methods that one reads before getting to that section.
8. The supplementary figures and main-text figures have some redundancy. I'd prefer a shorted supplement with figures that only illustrate truly supplementary information.
9. Supplement p. 6-7 – the story about the saddle-node bifurcations leading to a change the in neural network predictions is not well reflected in the main text. I would not have recognized the significance of bifurcations in the SSMs from the main text alone.
10. There is very little discussion about natural questions surrounding the emergence of chaotic dynamics. Is it helpful or

hurtful for learning if the RNN is chaotic? What happens to SSNs in the route to chaos? Is it just a successive loss of smoothness? And what are the implications for the data-driven approximations?
11. The supplementary methods concerning Lyapunov exponents and the implications of noise don't really seem very relevant for the rest of the manuscript. Do the lengthy calculations in the second half of the supplement lead to specific conclusions in the main text? And are they novel contributions here, or are they already reported in previous publications?

(Remarks on code availability)
The provided repository enables other researchers to explore the method to a reasonable extent.

Version 1:

Reviewer comments:

Reviewer #1

(Remarks to the Author)
I did read the response, and things have now been made clear. I am OK with the paper being published. I would be interested in a study (and maybe this is a future thing) of the SSMs of the effective equations, when they exist/can be derived, (and what they have to do with the SSMs of the "detailed" states).

(Remarks on code availability)

Reviewer #2

(Remarks to the Author)
The authors have thoughtfully revised the manuscript, addressing the points raised by the reviewers. I believe that the revisions significantly improved the presentation and broad audience appeal of the manuscript. Rather than presenting a striking novel contribution, the manuscript pedagogically and clearly contextualizes several slow-manifold observations in neural learning in the context of spectral submanifolds. I think it can successfully engage and educate an interdisciplinary audience, and I recommend publication.
I have a few final suggestions for the authors in their further revisions:
1. Fig. 1 – It would be helpful to label the input-on and input-off conditions in the panels so the conditions of each case are visually apparent. Also, the caption should note that the thin red/blue lines represent the unstable/stable manifolds.
2. In the discussion of Fig. 2, can you add some discussion about the readout that would result for the intermediate stable fixed points? Is it correct to interpret these fixed points as a state of non-confidence about the result?
3. Fig. 3 is not the most informative presentation in the present form. It assesses previous hypotheses about the learning dynamics, and notes the initial rate of change doesn't necessarily predict the final outcome, if I understand correctly. In the text, the authors note that the basins of attraction ultimately determine the decision. I think it would be better to depict the basins directly in some sort of plot, and note the previous hypotheses are inconsistent with the actual dynamics.
4. Sec. 2.2 – Are the "invariance error" and "prediction error" the same as the MFE and NMTE used previously? Also, is MTE in Fig. 1 caption supposed to be MFE? Since these scores are frequently referenced, I think you should include numbered equations in the methods with their definitions.
5. Fig. 4 – As in my comment about Fig. 1, it would be helpful to label the differing frequency conditions between the left and right panels on the plots.
6. P. 22, Sec. 4.1 end of third paragraph, the sentence "Additionally, in cases..." seems incomplete.
7. In relation to the comments from Reviewer #1 about effective population-level dynamics with steady states and my previous comments about applications to partial differential equations, the authors noted challenges related to the nonresonance conditions in the spectrum. This brings up interesting points for future considerations. Does the failure of the nonresonance conditions alone necessarily preclude the existence of a well-defined (unique and maximally smooth) SSM? For example, one particularly interesting case of an attracting invariant manifold in a population-level PDE model (along the lines that Reviewer 1 suggested) is the Ott-Antonsen manifold in synchronization literature. This manifold may be, in some sense, "attached" to the steady state representing the incoherent or synchronized states—can it also be interpreted as an SSM in the infinite-dimensional problem despite the failure of the nonresonance conditions?

(Remarks on code availability)

Open Access This Peer Review File is licensed under a Creative Commons Attribution 4.0 International License, which permits use, sharing, adaptation, distribution and reproduction in any medium or format, as long as you give appropriate credit to the original author(s) and the source, provide a link to the Creative Commons license, and indicate if changes were made.
In cases where reviewers are anonymous, credit should be given to 'Anonymous Referee' and the source.
The images or other third party material in this Peer Review File are included in the article's Creative Commons license, unless indicated otherwise in a credit line to the material. If material is not included in the article's Creative Commons license and your intended use is not permitted by statutory regulation or exceeds the permitted use, you will need to obtain permission directly from the copyright holder.
To view a copy of this license, visit https://creativecommons.org/licenses/by/4.0/

Reviewer #1:

We thank the Reviewer for the helpful comments and suggestions. Our itemized response is below, with the Reviewer's comments in highlighted boldface and the corresponding changes we have made in the text shown in italics.

1. **A quick "transitional" comment: I either disagree or misinterpret the statement on page 2 that "identifying such dynamical motifs requires accurate models of neural dynamics" – I am convinced that the phenomena are there for many, many different neuronal ODE models (I am sure we agree on that with the authors). The statement probably refers to the ability to quantitatively identify these behaviors.**

This is a good point. We now clarify in the text that we refer to the quantitative identification (and characterization) of these behaviors. In that sentence, we are referring to models of biological neural networks (which we currently lack in neuroscience).

We modified the sentence (lines 49-50) to: "*Quantitatively identifying and characterizing such dynamical motifs requires accurate models of neural population dynamics.*"

2. **This comment brings me to my second thought about the recommendation of the paper for publication. It is important to say, before I start, that what I am saying now is a personal opinion about the modeling of such physiological systems, that I can argue about, but since I am a "dynamical systems modeler with little experience in computational neuroscience" and not an actual neuroscientist, I can offer it only as a personal opinion.**
The authors work with actual steady states / limit cycles, and slow submanifolds of them, for systems of say O(100) differential equations. The important thing here for me is NOT the number 100 (it could be 1000, or 10,000) BUT the assumption that we have an ACTUAL steady state of that system, so we can compute its linearization (and then go on to computations of slow manifolds starting from its neighborhood). If that is accurate physically (if in neuroscience we have ACTUAL steady states of the equations for the neurons) then everything about the paper is OK.
If, however, as I suspect, we do NOT have actual steady states for large ensembles of neurons, but we rather have some sort of "statistical steady state" or "coarse grained steady state" or "statistical slow state" of the behavior, then I see a problem – the dynamical equations whose steady states we should work with are not the exact steady states of the 100 neurons, but rather some low-dimensional effective, coarse grained equations for the ensemble – a "closed" set of effective equations for the behavior obtained

somehow (theory-driven, data-driven) for the 100 or 1000 detailed dynamical neurons – which might never have an actual steady state at all, but rather, say, a local "ball" of small-scale messy/stochastic/chaotic behavior that can be thought of as a coarse-grained effective activity steady state.

The reviewer raises an important point: the existence of a steady state is indeed a key assumption. For SSM theory to apply, this steady state must be hyperbolic, but it does not necessarily need to be a fixed point. In practice, however, attaching SSMs to fixed points is far more feasible than attaching them to higher-dimensional or more complex steady-state solutions. Regarding the existence of (possibly unstable) fixed points in biological neural networks, we can only formulate hypotheses; RNN models themselves should be viewed as hypotheses about neural dynamics. Within this context, SSM reduction applied to RNNs can provide a more refined dynamical understanding and generate more specific, testable hypotheses, even without directly fitting SSMs to neural data. We share the reviewer's concern that such RNNs may ultimately be overly simplistic, even as proxy models of neural dynamics. However, there is some qualitative evidence that the nonlinear dynamics of RNNs can resemble those of neural circuits [Maheswaranathan, 2019; Barak, 2017; Mante, 2013; Sussillo, 2015], which explains the strong interest in these models. Our approach provides a systematic way to test whether such models capture meaningful aspects of neural dynamics.

We incorporate this point into the Discussion (lines 449-452)
"*The existence of a steady-state solution, a key assumption in SSM reduction, is well established for most mechanical systems. When modeling biological networks, instead, we can only make assumptions on the existence of (unstable) steady-state solutions to attach SSMs to.* "

3. **IF this option is true, then the paper does not discuss obtaining the effective coarser equations on which the SSM technology should be applied – it only talks about exact, detailed steady states/ limit cycles of the 100 or 1000 detailed neuron dynamical equations. If my view of the physiological situation is true, then a discussion of "how we get the equations on which to do SSM" is vital, and at the moment missing from the paper – and it should be meaningfully added. If the physiological situation involves actual steady states of 100 or 1000 neuronal model ODEs, or if the paper is talking about finite RNN model themselves, and not so much about the physiological situation, then the paper could be published as a dynamical reduction case study; yet I think the authors are more ambitious than that in their presenting and discussing connection with physiological neuroscience. SO: if there is a need for "effective" steady states and "effective" equations, this should be clarified and discussed. If I am wrong, and there is no need for "effective steady states" then the paper is OK as is.**

In the paper, we focus on the RNN dynamics, where this problem is less relevant because we have access to the equations of motion. However, we do believe that the methods could also be useful for analyzing neural data and, since we don't have foundational models of neuronal dynamics, the issue raised by the reviewer is very relevant to us and a research interest. In that case, whenever we are after an actual reduced model for the dynamics of biological neuronal networks, it is indeed very reasonable to think of them as being in a

“statistical slow state”, rather than converging to a fixed point or to a periodic/quasiperiodic orbit. There could be two strategies to go about this:

1. Considering the reduced models as arising from random dynamical systems [Arnold, 1998], as in the recent extension to SSM theory [Xu, 2024]. That of random dynamical systems is a formalism that allows us to maintain a deterministic core in the dynamics while accounting for (uniformly bounded) perturbations. In that case, fixed points of the “noise-free” dynamics are perturbed to trajectories that we can think of as statistical slow states.
2. As in [Liu, 2023], if we can hypothesize the existence of an unstable steady state for the system, it will have a so-called mixed-mode SSM, which might carry more complicated attractors (e.g., chaotic attractors).

We highlight this in the Discussion (lines 455-460)
“*This setting (...) preserves a nonlinear deterministic core while explicitly accounting for biologically plausible fluctuations. The special solutions we can attach SSMs to are then not exact steady states. Instead, they become random, time-dependent anchor states arising from uniformly bounded perturbations to the exact steady states of the autonomous dynamics.”*

Reviewer #2:

We thank the Reviewer for the helpful comments and suggestions. Our itemized response is below, with the Reviewer’s comments in highlighted boldface and the corresponding changes we have made in the text shown in italics.

1. **The content and presentation style are somewhat unusual for a broad-audience journal. Broadly, I felt that the methods and supplementary information together were basically a long-format technical article, and the main text was an effort to compress that information into the journal’s more constrictive article format. Consequently, the main text may be hard to follow for an average reader who lacks a strong dynamical systems background.**

We thank the reviewer for this feedback. Given the limited space, we could not include extensive explanations of dynamical systems theory to help the broader audience. However, to address the reviewer’s concern, we added explanations of key points in the main manuscript that target non-expert readers:

- (Introduction, lines 66-74): “*Central to our approach is the recent theory of Spectral Submanifolds (SSMs), which are ubiquitous and robust low-dimensional, attracting invariant manifolds that originate from steady state solutions along eigenspaces of the locally linearized dynamics (see [SSM Review] for a general introduction, applications, and further references). Invariant manifolds are sets in the phase space that have a manifold structure and are mapped to themselves by the system’s evolution map, the flow map. These manifolds are attracting if the trajectories*

converge to them asymptotically, while they are robust if they persist as an invariant set under small perturbations of the system, and smoothly depend on them."

- (Results, lines 123-125): "*A spectrum is nonresonant if no eigenvalue can be written as a linear combination of the other eigenvalues with positive integer coefficients. (...) While the existence of SSMs is deduced from the linearized spectrum at a steady state, these invariant manifolds do extend beyond the domain of linearization and hence can carry characteristically nonlinear dynamics*."
- (Results, lines 147-148): "*As a result, parameters are explicitly present in the SSM and reduced dynamics equations, enabling us to clearly understand their role.*"
- (Results, lines 362-364): "*A continuum of fixed points, however, is inherently non-robust and can be destroyed by small perturbations to the neuronal dynamics like, for example, the ones coming from the biological variability of the neurons, or small variations in experimental conditions.*"
- (Methods, lines 484-485): "*One can easily check this fact by looking at the degree of smoothness at zero of trajectories of a linear system that do not coincide with the eigenspaces.*"
- (Methods, line 504): "*no low-order (with coefficients m_j such that $2\leq \sum_{i=1}^k m_k \leq \sigma(E)$) integer linear combination of eigenvalues $\sum_{j = 1}^k m_k \lambda_k $ within \(E \) should coincide with an eigenvalue outside of \(E \).*"
- (Methods, lines 531-532): "*where $Dh(0)$ is the differential of h at 0 with respect to (η_1, η_2)*".

2. **Another small complaint that I have is that the class of "recurrent neural networks" encapsulated by the coupled ODEs in Eq. (14) is rather specific to the dynamical systems framework, which might be considered unconventional. Many artificial neural networks do not really have a temporal variable at all. The models and slow point views described in the introduction on p3 are more narrowly focused on a specific class of biological systems and neuronal dynamics, so much so that I felt that a more descriptive title for the manuscript would be "Data-Driven Reduced Modeling of Recurrent Neuronal Dynamics." An overly cynical reader may conclude that the manuscript is the work of mathematicians who cherry-picked applied research to find evidence of impactful work that would support their preferred theory.**

Indeed, the term RNN refers to a broader class of artificial neural networks that includes models that may not be framed as dynamical systems. The RNNs we use in the paper are primarily used in computational neuroscience as proxy models for neuronal networks. In this context, we do not study a "toy problem", as RNNs with this specific form, whose technical name would be *Neural ODEs*, have emerged as a key tool for generating hypotheses about neural computations [Maheswaranathan, 2019; Kanwisher, 2023; Driscoll, 2024; GR Yang, 2020; Mante, 2013; Sussillo, 2015; Durstewitz, 2023; Song, 2016]. Following the reviewer's suggestion, to address any misunderstandings we propose to change the title to: "Data-Driven Reduced Modeling of Neural Dynamics".

We change the term RNN with the term Neural ODEs in the abstract and, in the Introduction, we have added (lines 80-81): "*We demonstrate the power of data—driven SSM—based modeling for neural dynamics using simulation output data from neural ordinary differential*

equations, also referred to as recurrent neural networks (RNNs)\footnote{We will use the term RNN in the rest of the paper, as it is clear from the context that we mean the specific class of RNNs identifiable with neural ODEs}"

3. **I was left a little skeptical about how impactful the results would be to a broader audience, including the few among the developers of the models studied.**

We believe this work is important for neuroscience because, with the availability of new datasets comprising large-scale recordings of neural dynamics, dynamical systems-based reduction methods have become increasingly popular. RNNs have become a widely used tool in neuroscience, not only for reproducing neural population dynamics but also for generating hypotheses about mechanisms and computation (see references above). Since RNNs themselves do not explain the observed behavior of neurons, the goal is to abstract the essence of the computations, focusing on the interactions between inputs and recurrent dynamics. In this sense, the description of reduced models is more parsimonious.

Most dynamical systems concepts have been used rather loosely in the field, and the appropriate formal tools are often missing. One of the aims of the paper is to show that they can be used and how.
For this reason, the educational aspect is also important: the effort is to inject new tools, beyond fixed points and linearized dynamics, into the neuroscience community.

We emphasize and clarify this point in the revised text: (lines 81-86): "*RNNs trained on specific behavioral tasks have emerged not only as a key tool for reproducing the features of neural dynamics in brain recordings, but also for generating hypotheses about the neural computations implemented by the underlying neural circuits. The goal is to abstract the essence of the computations, focusing on the interactions between inputs and recurrent dynamics [Maheswaranathan, 2019; Kanwisher, 2023;* Mante, 2013; Sussillo, 2015; Durstewitz, 2023; Song, 2016]*]* "

We further improved the educational aspect by making the content more accessible with the modified text, as discussed in point 2.

4. **Nevertheless, it stirred some inspirational thoughts in my mind. For example, in the study partial differential equations, symmetries in the jet space approach [Olver, Applications of Lie groups to differential equations. Springer, 1993] give rise to self-similar solutions that generalize fixed points in an appropriate coordinate system of invariants (see, e.g. the rather obscure and inconsequential [Nicolaou, Symmetry and variational analyses of fluid interface equations in the thin film limit, 2017]). Could the SSM approach say anything broadly about the development of turbulence, or could the data-driven approximations give rise to new approaches?**

We thank the reviewer for this insightful comment, which may suggest potential future applications in neuroscience, particularly in contexts where more appropriate neuronal network models account for the network's spatial structure. The symmetries in the jet space

approach is appealing, as, to our understanding, it allows one to translate the geometrical analysis of the phase space of ODEs (seen as time-translation-invariant PDEs with time as the only independent variable) to the phase space of PDEs. Importantly, this construction does not rely on discretization but only on the theorem on the analytic existence and uniqueness of solutions. Some comments:

- Because of the resonance conditions, it is not trivial to prove the existence of SSMs in infinite-dimensional spaces. However, [Buza, 2023] proved that they exist for the Navier-Stokes equations.
- Based on the construction in [Nicolau, 2017], given a PDE with m independent variables and n_s symmetries, self-similar solutions are, to our understanding, generalizations of fixed points in the (infinite-dimensional) extended *phase space* that reduce to $(m - n_s)$-dimensional surfaces in the phase space, once the symmetries have been quotiented out. In general, it would be possible but practically not very feasible to attach an SSM to such solutions if $(m - n_s)$is bigger than one.
- However, for pipe flows with a periodic domain, in [Kaszas, 2024] the translational symmetry of the Navier-Stokes equation is exploited to attach a 2-dimensional SSM to a saddle-type edge state. The stable manifold of the edge state acts as a boundary that divides trajectories that converge immediately to the laminar state from those that exhibit transient behavior. In this paper, the SSM is constructed in a data-driven fashion from solutions of the Navier-Stokes equations via discretization.

This discussion is outside the scope of our paper, but we would be happy to continue it in another context.

We have added the reference to [Kaszas, 2024] to the revised Method section (lines 613-615): "*Similarly, the existence of an unstable steady state solution, the edge state, is used in [Kaszas, 2024] to describe the transition to turbulence in periodic pipe flows*."

5. **What exactly is the goal of producing a reduced-order model for an RNN? To replace the RNN with a less expensive surrogate? Or to make some more interpretable model to show how learning works?**

Within the scope of the RNNs used in our paper, the reduced dynamics elucidate the dynamical principles underlying learning and computation. They serve the purpose of identifying "abstract" mechanisms, finding the most parsimonious low-dimensional description of the dynamics that still captures the underlying computation. Such dynamical descriptions, in turn, lead to hypotheses about the effect of causal perturbations that are becoming experimentally testable [Vinograd, 2024]. This perspective is also relevant in emerging areas such as computational psychiatry, where it has been suggested [Murray, 2018] that such reduced descriptions may have practical diagnostic value by distilling key differences in neural dynamics and function between patient groups.

In a broader context, RNNs are used, for example, as models in control circuits. In that case, faster simulations become very important.

We emphasize this in the revised text: (lines 434-443) "*For RNNs used as models for neural population dynamics, the key advantage of model reduction is interpretability: it provides insight into the structural organization of the learned dynamics. Reduced models help identify abstract mechanisms, namely, the most parsimonious low-dimensional description of the dynamics that still captures the underlying computation [Vinograd, 2024]. This perspective is becoming relevant in emerging areas such as computational psychiatry [Murray, 2018], where such reduced descriptions may have practical diagnostic value. For RNNs used in broader contexts (e.g., control circuits), reduced-order models can serve as less expensive surrogates to reduce computational cost and enable faster simulations*"

6. **Figure captions in the main text are huge, and panels aren't referenced individually and in order that they appear in the main text, making them a little impenetrable. Similarly, I'd prefer that the sections of the supplement and the methods were referenced in the main text where first relevant, so that it was possible to read just the main text alone to understand the most important message and reference methods and the supplement only if/when desired.**

To improve readability and avoid redundancies, we reorganized the manuscript as follows:

- More careful figure referencing
- All references to figures in the Supplements were removed
- Fig. S2 was moved from the Supplements to the main body (section 2.1, Fig.2). Fig.2 now consists of the bottom most part of the (former) Fig.1 and the former Fig. S2.
- Reduced Fig.1 caption size
- Modified Supplementary Information " Context-Dependent Decision-Making RNN: Details on the Reduced Dynamics" to "Context-Dependent Decision-Making RNN: Uniformly Bounded Noise"
- Reordering of the Supplementary Information (what appears first in the main body is first in the supplementary information:
    S1: Finite-Time Lyapunov Exponent (FTLE)
    S2: Context-Dependent Decision-Making RNN: Parameter Dependent SSMs
    S3: Context-Dependent Decision-Making RNN: Uniformly Bounded Noise
- New referencing of Supplementary Methods (SM)

These changes also helped us to address points 11-12-13 raised later by the reviewer.

7. **a. For readers hoping to use the SSMLearn package without strong dynamical systems expertise, more practical guidance would be helpful for a broad-audience publication.**

To disseminate SSM theory and ensure the correct use of the SSMLearn package, the Haller group has made several online resources available for practical know-how. In particular, on the GitHub page of the group, there are fully dedicated tutorial slides https://github.com/haller-group/SSMLearn/blob/main/docs/SSMLearn_tutorial.pdf and practical considerations slides https://github.com/haller-group/SSMLearn/blob/main/docs/Practical_considerations.pdf. These serve as support for the worked-out examples in the SSMLearn repository and for the book that summarizes all SSM theory and related applications [Haller, 2025] that the group recently published.

We have added references to these resources to the Method section of the new version of the manuscript (lines 644-651): "*To ensure the correct use of the SSMLearn package, several online resources are available for practical guidance. In particular, the reader can find fully dedicated tutorial slides at \url{https://github.com/haller-group/SSMLearn/blob/main/docs/SSMLearn_tutorial.pdf} and practical considerations slides at \url{https://github.com/haller-group/SSMLearn/blob/main/docs/Practical_considerations.pdf}. These serve as support for the worked-out examples in the SSMLearn repository and for the book that summarizes all SSM theory and related applications \cite{SSMReview}.*"

7. b. How does one select the dimension d of the manifold and the order of the Taylor series approximation? Can you provide a score for the fit on a test dataset and a Pareto-front or AIC criterion for the selection?

The choice of order d of the manifold is a principled choice based on the comparison of the timescales of the problem at hand with the timescales of the system that we want to model. SSMs of any dimension exist, but to capture a system's core dynamics, we most often want to choose the slow ones, which persist after the transients (which we may define differently depending on the problem) have decayed. One can read this information from the linearization spectrum and examine the spectral gaps between the (real parts of the) eigenvalues.

Some remarks on the choice of the order for the Taylor expansion of the SSM/its reduced dynamics: there is currently no automated pipeline for this choice, but the idea is that, within the radius of convergence of the Taylor expansion around the fixed point, if the SSMs/ROMs are analytic, higher-order approximations should yield better results. Since, however, we generally don't know the convergence domain and want to reduce computational burden, we stop at the lowest order of the expansion that captures the core dynamics. A score for this fit is usually the mean-square distance with test trajectories: we use the manifold fitting error (MFE) for the SSM and the normalized mean trajectory error (NMTE) for the reduced dynamics, as referenced in the paper. These metrics already account for the fact that, at higher orders, outside the convergence domain, the approximation is less accurate than at lower orders, so it is not really a tradeoff between the complexity of the model and the "goodness" of the results (as in the AIC criterion). We currently do not have a Pareto-front criterion for selection, as it depends heavily on the specific use case. One could plot, say, the NMTE, and the computation time of the reduced dynamics or the integration time for the reduced equation.

We incorporate the remark on the choice of the order for the Taylor expansion in the method section (lines 672-684): "T*here is currently no automated pipeline for selecting the order of the Taylor expansion approximating the SSM or the reduced dynamics. For analytic SSMs, within the radius of convergence of the Taylor expansion around the fixed point, higher-order approximations should yield better local results. Since, however, we generally don't know the convergence domain, we stop at the lowest order of the expansion that captures the core dynamics. We usually evaluate the approximations through the mean-square distance with test trajectories: we use the manifold fitting error (MFE) for the SSM and the normalized mean trajectory error (NMTE) for the reduced dynamics. The MFE measures the mean-square distance between the lifts of reduced test trajectories and the SSM and the*

actual test trajectories. If there were no noise and the SSM model were exact, this distance should be zero. Similarly, the NMTE measures the distance between the reduced model's predictions, given test initial conditions, and the actual test trajectories."

8. **Fig. 5: are there typos in the caption? The sine wave is on the right and the context choice is left?**

Indeed, we fixed this.

9. **The future tense at the end of the methods sections, e.g. pg 24 "We will treat this discontinuous…" is odd. If the supplement is truly supplementary, the reader should be able to read the main text without worrying about what will be done in the supplement. It would be better to note that "We treat this discontinuous…" as a part of the discussion in the main text.**

This is a good point. We removed the sentence from the method section. The strategy we use to treat the discontinuous model has already been noted in the main text.

10. **Creating a recurrent neural network to generate a sine wave is certainly an odd goal without some context. It only takes two real variables to describe a simple harmonic oscillator—why introduce a bulky neural network?**

We agree, some context is needed. In a way, we could make the same remark about the RNN for context-dependent decision making. As SSM reduction shows, a minimal model with the same computational features would consist of a 1-dimensional system with three fixed points, two stable and one unstable, and a dependency on parameters that accounts for context-dependent modulation. The idea underlying training bulky RNNs to perform relatively simple tasks is that, in real life, the same simple tasks are performed by complex, high-dimensional networks of biological neurons. The ODE for the RNN is a simplified *rate model* for the neural network that performs the task.
In the case of the oscillatory dynamics of the "sine-wave generator" RNN, the goal is to achieve periodic behavior given a high-dimensional network of neurons. The idea comes from the fact that the primary motor cortex in primates (M1) seems to generate oscillatory dynamics at different frequencies, from which movements can be generated (see [Sussillo, 2015]).

We have added this context to the text in the Method section (<u>lines 736-741</u>):
"*The behavior implemented in this case is not linked to a specific experiment but rather a general model of oscillatory responses in a neuronal network. It is relevant to the study of the primary motor cortex in primates, where a high-dimensional network of interconnected neurons appears to generate oscillatory dynamics at different frequencies during movement onset and muscle contraction.*"

11. **Supplement, p3: "inverient" is a typo. Also, the paragraph before S1 is pretty repetitive given the main-text methods that one reads before getting to that section.**

Thanks, we fixed the typo. We also removed all redundancies with the main-text methods in S1. We only left the discussion about the dynamics of the noisy RNN (Hence, the change in the title: "*Context-Dependent Decision-Making RNN: Uniformly Bounded Noise*"

12. **The supplementary figures and main-text figures have some redundancy. I'd prefer a shorted supplement with figures that only illustrate truly supplementary information.**

We realize that some information was repeated between section 2.1 and (former) S1. We removed all the redundant parts in that supplementary section "Context-Dependent Decision-Making RNN: Details on the Reduced Dynamics" along with its figures (hence the change in the title, see answer to point 11). The relevant figure (former S2) was moved to the main body, in section 2.1.

13. **Supplement p. 6-7 – the story about the saddle-node bifurcations leading to a change the in neural network predictions is not well reflected in the main text. I would not have recognized the significance of bifurcations in the SSMs from the main text alone.**

We have added the figure with the bifurcation diagram in the main text, section 2.1, Fig. 2. We also now mention the cascade of saddle-node bifurcations in section 2.1 (lines 224-232): *"We note that, even though the two stable fixed points corresponding to the two choices remain fixed, the number and location of the other fixed points change with the parameter. In particular, four saddle-node bifurcations occur within a small interval around s2 = 0, as illustrated in the bifurcation diagram in Fig. 2 (bottom left and right). It is through this cascade of saddle-node bifurcations that the system adapts to favor the convergence to the stable fixed point that yields the correct readout (positive for positive values of s2 and vice versa) along the branches of the 1D unstable manifold of an unstable fixed point (see Supplementary Material S2 for a more detailed discussion)"*

14. **There is very little discussion about natural questions surrounding the emergence of chaotic dynamics. Is it helpful or hurtful for learning if the RNN is chaotic? What happens to SSMs in the route to chaos? Is it just a successive loss of smoothness? And what are the implications for the data-driven approximations?**

The dynamics of the RNNs under study are far from being chaotic. However, for future work, a discussion of capturing chaotic dynamics is very useful, especially for applications to biological data. In [Liu, 2023], the dimensionality of a chaotic system is reduced via an SSM-reduced model. In that case, the SSM is not attached to the chaotic attractor itself, but it still carries the attractor and the chaotic dynamics.

The key requirement is the presence of an unstable fixed point in the phase space of the system: the unstable fixed point has an SSM that coincides with its unstable manifold, and mixed-mode SSMs consisting of the unstable manifold and slow, stable SSMs of different dimensions. Depending on the Hausdorff dimension of the chaotic attractor, high-dimensional enough mixed-mode SSMs (or even the unstable manifold, if its dimension is large enough compared to the dimension of the attractor) will carry the attracting set of the chaotic attractor.

As a consequence, the reduced model on the SSM accounts for the chaotic dynamics. Therefore, there's no loss of smoothness involved: if the system is smooth, the SSM and its reduced dynamics remain smooth (this would not be the case if we anchored the SSM to the chaotic attractor itself). The only difference for the data-driven approximation is that, for test trajectories converging to the chaotic attractor, we cannot hope for deterministic predictions. Given small errors in the initial conditions, chaotic trajectories are not predictable. One can, however, predict the statistics of the ergodic measure of the chaotic attractor using reduced dynamics and evaluate the reduced model on those statistics. Anchoring a parameter-dependent SSM to the unstable fixed point can capture the transition to chaos via a parameter-dependent reduced model.

We have added the reference to [Liu, 2023] in the Method section (lines 601-613): "*We can also develop a strategy to exploit the smooth dependence of SSMs on parameters to obtain a reduced-order model of the transition to chaotic dynamics. The key requirement for this strategy to work is the existence of a persistent, unstable fixed point in the phase space of the system that contains the bifurcating attractor. The unstable fixed point has an SSM that coincides with its unstable manifold, and mixed-mode SSMs consisting of the unstable manifold and slow, stable SSMs of different dimensions. Based on the Hausdorff dimension of the chaotic attractor, we can choose a high-dimensional enough, parameter-dependent SSM attached to the unstable fixed point. This SSM will carry the bifurcating attracting set transitioning to a chaotic attractor; the parameter-dependent reduced model on the SSM will account for the transition to the chaotic dynamics. See [Liu, 2023] for examples of SSM-reduced models of chaotic systems with an unstable fixed point in their phase space*"

15. **The supplementary methods concerning Lyapunov exponents and the implications of noise don't really seem very relevant for the rest of the manuscript. Do the lengthy calculations in the second half of the supplement lead to specific conclusions in the main text? And are they novel contributions here, or are they already reported in previous publications?**

We chose to include the FTLE analysis because it provides a principled way to find the boundary of the domains of attraction of the stable fixed points. While FTLEs are widely used in dynamical systems and fluid dynamics, they are much less common in the neuroscience literature. However, in the neuroscience literature, several decision-making experiments involving multistability could benefit from this analysis. One of our goals in including it is educational: we want to introduce and demonstrate the usefulness of dynamical systems-based diagnostics for analyzing RNN dynamics in a neuroscience context. Because this analysis is somewhat complementary to the main narrative, and due to space considerations, we chose to present it in the Supplementary Methods rather than in the main text.

The implications of noise are very relevant for future applications to biological data. Regarding the calculations in the second half of the supplement, the formulas for anchor trajectories and time-dependent spectral submanifolds (SSMs) are based on general results derived in [Haller, 2024]. In the supplement, we specialize these formulas for our specific system to make the construction explicit and favor reproducibility of our results (one could check our calculations step by step). One could read those formulas from the publicly available code, but that would not be as straightforward.

We thank the Reviewer for the final comments and suggestions. Our itemized response is below, with the Reviewer's comments in highlighted boldface and the corresponding changes we have made in the text shown in italics.

1. Fig. 1 – It would be helpful to label the input-on and input-off conditions in the panels so the conditions of each case are visually apparent. Also, the caption should note that the thin red/blue lines represent the unstable/stable manifolds.
Fig. 3 is not the most informative presentation in the present form. It assesses previous hypotheses about the learning dynamics, and notes the initial rate of change doesn't necessarily predict the final outcome, if I understand correctly. In the text, the authors note that the basins of attraction ultimately determine the decision. I think it would be better to depict the basins directly in some sort of plot, and note the previous hypotheses are inconsistent with the actual dynamics.
Fig. 4 – As in my comment about Fig. 1, it would be helpful to label the differing frequency conditions between the left and right panels on the plots.

We modified figures 1, 3 and 4 accordingly.

2. In the discussion of Fig. 2, can you add some discussion about the readout that would result for the intermediate stable fixed points? Is it correct to interpret these fixed points as a state of non-confidence about the result?

We added the sentence: "*This cascade of bifurcations occurs, however, for sensory inputs close to zero. In the original experiment of [Mante, 2013], the network would be, for the sensory input off-condition, always initialized close to zero. Therefore, trajectories would converge always to the same fixed point (in brackets in Fig. 2), which coincides with the stable fixed point on the right hand side of Fig. 1.*"

3. Sec. 2.2 – Are the "invariance error" and "prediction error" the same as the MFE and NMTE used previously? Also, is MTE in Fig. 1 caption supposed to be MFE? Since these scores are frequently referenced, I think you should include numbered equations in the methods with their definitions.

We removed the ambiguity by consistently using "MFE" and "NMTE" instead of "invariance error" and "prediction error". We also added the definitions in the methods 4.2 and referenced them in the main text accordingly.

4. **P. 22, Sec. 4.1 end of third paragraph, the sentence "Additionally, in cases..." seems incomplete**

We rephrased as: "*Specifically, varying the additive parameters in the input vector can modify the phase space geometry by shifting the fixed point locations and altering the sizes of their domains of attraction. Additionally, when parameter values are close to bifurcation values, this can lead to changes in the stability of the fixed points or to their disappearance*."

5. In relation to the comments from Reviewer #1 about effective population-level dynamics with steady states and my previous comments about applications to partial differential equations, the authors noted challenges related to the nonresonance conditions in the spectrum. This brings up interesting points for future considerations. Does the failure of the nonresonance conditions alone necessarily preclude the existence of a well-defined (unique and maximally smooth) SSM? For example, one particularly interesting case of an attracting invariant manifold in a population-level PDE model (along the lines that Reviewer 1 suggested) is the Ott-Antonsen manifold in synchronization literature. This manifold may be, in some sense, "attached" to the steady state representing the incoherent or synchronized states—can it also be interpreted as an SSM in the infinite-dimensional problem despite the failure of the nonresonance conditions?

We thank the reviewer for raising this interesting question.

- As discussed in [Haller and Ponsioen, 2016], SSMs can be thought of as the smoothest nonlinear continuations to eigenspaces. While the standard nonresonance conditions are not always necessary to prove SSM existence (see [Buza, 2023]), their failure forbids the systematic order-by-order construction of polynomial expansions of SSMs and the reduced dynamics on them.
- To our understanding, the Ott–Antonsen manifold arises in the infinite-population limit of coupled oscillator systems, and is an infinite-dimensional invariant manifold for the dynamics of the distribution function in the infinite-dimensional space of phase and frequency distributions. It is anchored to a non-hyperbolic state with a continuous neutral spectrum; it is attracting (when it is) by Landau damping rather than spectral contraction. The reduced dynamics arise from defining "order parameters" that describe the macroscopic behavior of the infinite-dimensional system (as common in mean-field/renormalization frameworks).
- Although there are clear conceptual links between model reduction, normal-form theory, and the renormalization-group/mean-field ideas used to describe collective phenomena and phase transitions (this link is even made explicit in [DeVille, 2008], we would hesitate to identify the Ott–Antonsen manifold as an SSM, not because of the failure of nonresonance conditions but because of its "nonspectral" nature. However, the existence of low-dimensional structures arising from mean-field/renormalization constructions suggests potentially interesting investigations of this link via SSM theory, beyond the scope of the present work.

-
